# Heterogeneity of Polyneuropathy Associated with Anti-MAG Antibodies

**DOI:** 10.1155/2015/450391

**Published:** 2015-05-06

**Authors:** Laurent Magy, Raphaël Kaboré, Stéphane Mathis, Prisca Lebeau, Karima Ghorab, Christiane Caudie, Jean-Michel Vallat

**Affiliations:** ^1^Department of Neurology, Centre de Référence “Neuropathies Périphériques Rares”, CHU Limoges, 2 Avenue Martin Luther-King, 87042 Limoges, France; ^2^Department of Neurology, CHU Poitiers, University of Poitiers, 2 Rue de la Milétrie, 86021 Poitiers, France; ^3^Centre de Biologie et de Pathologie Est, Hospices Civils de Lyon, 69000 Lyon, France

## Abstract

Polyneuropathy associated with IgM monoclonal gammopathy and anti-myelin associated glycoprotein (MAG) antibodies is an immune-mediated demyelinating neuropathy. The pathophysiology of this condition is likely to involve anti-MAG antibody deposition on myelin sheaths of the peripheral nerves and it is supposed to be distinct from chronic inflammatory demyelinating neuropathy (CIDP), another immune-mediated demyelinating peripheral neuropathy. In this series, we have retrospectively reviewed clinical and laboratory findings from 60 patients with polyneuropathy, IgM gammopathy, and anti-MAG antibodies. We found that the clinical picture in these patients is highly variable suggesting a direct link between the monoclonal gammopathy and the neuropathy. Conversely, one-third of patients had a CIDP-like phenotype on electrodiagnostic testing and this was correlated with a low titer of anti-MAG antibodies and the absence of widening of myelin lamellae. Our data suggest that polyneuropathy associated with anti-MAG antibodies is less homogeneous than previously said and that the pathophysiology of the condition is likely to be heterogeneous as well with the self-antigen being MAG in most of the patients but possibly being another component of myelin in the others.

## 1. Introduction

Ten percent of patients with a polyneuropathy of unknown cause have a monoclonal gammopathy [1]. Most of these patients have an IgM dysglobulinemia and around 70% of those have anti-myelin associated glycoprotein (MAG) antibody detected by enzyme-linked immunosorbent assay (ELISA). Indeed, it has long been demonstrated that MAG behaves as a self-antigen in patients with polyneuropathy and IgM monoclonal gammopathy [2]. In the past 25 years, numerous series have described anti-MAG neuropathy as a homogeneous entity [3, 4]. The clinical picture of the disorder usually consists of a chronic sensory polyneuropathy with ataxia and tremor of progressive worsening. Motor involvement, if present, usually occurs lately in the course of the disorder [5]. Nerve conduction studies display a demyelinating pattern with distally accentuated slowing of motor conduction, no conduction block, and a severe reduction of sensory nerve action potentials (SNAPs) [6]. When nerve biopsy is performed, it shows signs of demyelination on semithin sections and teased fiber studies, and electron microscopic examination usually displays the classic pattern of widening of myelin lamellae (WML), which is considered the pathological hallmark of the disease [7]. This latter feature corresponding to deposits of the monoclonal IgM on myelin sheath distinguishes pathologically anti-MAG neuropathy from chronic inflammatory demyelinating polyradiculoneuropathy (CIDP) [8].

In the present study, we show that anti-MAG neuropathy is indeed a heterogeneous disorder as demonstrated by careful clinical, electrophysiological, and neuropathological analysis. We discuss the potential reasons for this heterogeneity and its therapeutic implications.

## 2. Patients and Methods

The data from all patients with a polyneuropathy associated with an IgM monoclonal gammopathy and anti-MAG antibodies seen in our neurology department over the previous 25 years were retrospectively reviewed.

### 2.1. Clinical Findings

Age, gender, and duration of symptoms at the time of diagnosis were extracted from the medical charts. Based on clinical evaluation, patients were classified as having pure sensory neuropathy, ataxia with sensory neuropathy, and sensorimotor neuropathy. Ataxia was considered if patients had a positive Romberg sign, subjective impression of balance loss, and visible balance disturbance when walking. A sensorimotor neuropathy was defined by the presence of sensory loss on clinical examination and motor weakness at 4 or less on the Medical Research Council (MRC) scale in any limb segment (except if patients had weakness only in toe extensors).

### 2.2. Electrodiagnostic Studies

At the time of referral, 56 (93%) patients had nerve conduction studies performed in our neurophysiology department as described [9]. Bilateral motor conduction studies of median, ulnar, peroneal, and tibial nerves and sensory conduction studies of sural, median, and ulnar nerves were performed. The nerve conduction data were considered sufficient for analysis when at least 2 motor nerves and one sensory nerve were examined in the lower limbs and 2 motor nerves and 2 sensory nerves in the upper limbs. Partial conduction block was defined by a reduction of compound muscle action potential (CMAP) by proximal stimulation of at least 50% in the lower limb and at least 30% in the upper limb. Temporal dispersion was defined by a lengthening of CMAP of at least 30% by proximal stimulation. The terminal latency index (TLI) was calculated for the median and ulnar nerves as described [10]. For the purpose of this study, patients were retrospectively classified as having typical anti-MAG neuropathy: a grossly symmetric demyelinating neuropathy with distally predominant demyelination based on low terminal latency indexes, a severe decrease of sensory nerve action potential (SNAP) amplitudes in the lower limbs, and no conduction block or temporal dispersion [11], or a CIDP-like pattern: fulfillment of the EFNS/PNS criteria for CIDP [12], normal TLI in ulnar and median nerves and/or presence of conduction block and/or temporal dispersion in at least one nerve, and/or normal or near normal SNAPs in the lower limbs.

### 2.3. Biological Studies

All patients had IgM monoclonal gammopathy determined by serum immunoelectrophoresis or immunofixation. Search for anti-MAG antibodies was done several years ago for 4 patients by immunochromatography. Sera from most of the patients were tested for anti-MAG antibodies by ELISA (Bühlmann laboratories AG, Schonenbuch, Switzerland). The cut-off positive value was considered above 1000 BTU (Bühlmann Titer Unit) according to the manufacturer's instructions [13]. Other sera were tested in the center of biology and pathology (Hospices Civils de Lyon) for anti-MAG antibodies and for antibodies to sulfate-3-glucuronyl paragloboside. To confirm anti-myelin activity, we performed indirect immunofluorescence with the sera from 45 patients on normal human peripheral nerve as described earlier [14]. In 15 patients, we tested for the presence of anti-glycolipid antibodies as described previously [15].

### 2.4. Nerve Biopsy

Forty-three of the 60 patients underwent nerve biopsy after informed consent. Depending on clinical and/or neurophysiological involvement, the sural, superficial peroneal or sensory branch of a radial nerve was taken. One fragment was fixed in 10% formaldehyde and embedded in paraffin. Sections were stained using conventional methods. Other fascicles were fixed in buffered glutaraldehyde and then embedded in Epon. Several blocks were used for semithin sections subsequently colored with toluidine blue. Ultrathin sections were prepared as described [14] and viewed in a JEOL electron microscope. A third fragment was frozen for direct immunofluorescence (DIF) using specific antisera for IgG, IgA, IgM, lambda and kappa light chains, and C3d conjugated to fluorescein, to reveal immunoglobulin deposits on the patients' nerves.

### 2.5. Statistical Analysis

The clinical and laboratory data were collected and analyzed with the SPSS 8.0 software. Qualitative data were expressed as percentages with their 95% confidence interval and quantitative data were expressed as mean ± SD. Statistical significance of differences between categorical variables was determined by means of *χ* ^2^ or Fisher exact test as appropriate and for differences between continuous variables by means of Student's *t*-test. Statistical significance for all analyses was defined as *p* < 0.05.

## 3. Results

The main clinical and laboratory data are summarized in Table 1.

### 3.1. Demographic and Clinical Data

There were 60 patients (43 males: 72%). Mean age at study was 67 ± 10 years (range 46–87) and mean duration of symptoms at diagnosis was 35 ± 44 months (range 2–204). Nineteen (32%) patients had the distal acquired demyelinating symmetrical (DADS) phenotype [16]. Symptoms and signs in these patients were mostly sensory and proximal segments of limbs were never involved by sensory and/or motor signs. These patients had usually numbness or tingling limited to the feet and prominent ataxia and 3 of them had additional pain. Tendon reflexes were abolished at least in the lower limbs in these patients. Seventeen (28%) patients had isolated distal numbness or pain without ataxia. Pain had the classical characteristics of neuropathic pain [17] in these patients (mainly burning, squeezing, and painful cold) and was a prominent symptom in seven of those who used various analgesics (clonazepam, gabapentin, amitriptyline, and pregabalin). Physical examination showed distal hypesthesia and tendon reflexes were variably impaired in this subgroup of patients (ranging from diminished Achilles reflexes to abolition of reflexes in all four limbs). 32 (53%) patients had variable degrees of motor weakness leading to severe impairment in a minority of them. Weakness was usually distal although 2 patients had prominent proximal weakness. Mean duration of symptoms at diagnosis was 45.6 months in patients with sensorimotor neuropathy and 31.9 months in other patients although this difference did not reach significance. The presence of any weakness was not statistically linked with the duration of symptoms when comparing patients with duration of disease below or above two years.

### 3.2. Nerve Conduction Studies

Fifty-six patients had exploitable nerve conduction studies. 37 (66%) patients had the typical anti-MAG pattern as defined above [11]. Most of them had a severe reduction of SNAPs in all four limbs, a distal pattern of demyelination as defined by low TLIs in at least one nerve, and no conduction block. 17 (30%) patients had nerve conduction studies consistent with CIDP [12]. Most of these patients had conduction block and/or marked temporal dispersion and some had normal or near normal SNAPs in the lower limbs. The two remaining patients had a sensory axonal neuropathy. There was no correlation between the different clinical subtypes and the electrodiagnostic pattern of nerve involvement.

### 3.3. Biological Data

All patients had IgM monoclonal gammopathy and anti-MAG antibodies. For 51 (85%) patients, the diagnosis was of a monoclonal gammopathy of undetermined significance (MGUS) although 4 patients had Waldenström's macroglobulinemia, 2 patients had non-Hodgkin's malignant lymphoma, and 3 patients had chronic leukemia. One patient with chronic lymphocytic leukemia had epineurial B cell infiltrates suggestive of lymphomatous transformation (see below). The titer of anti-MAG antibodies was highly variable among patients, ranging from 1111 BTU to > 70000 BTU (Table 1). Although quite strongly correlated with the neuropathological pattern and the electrodiagnostic findings, the biological data were not correlated with the clinical pattern as defined above.

### 3.4. Neuropathology and Indirect Immunofluorescence Studies

**Figure 1:**
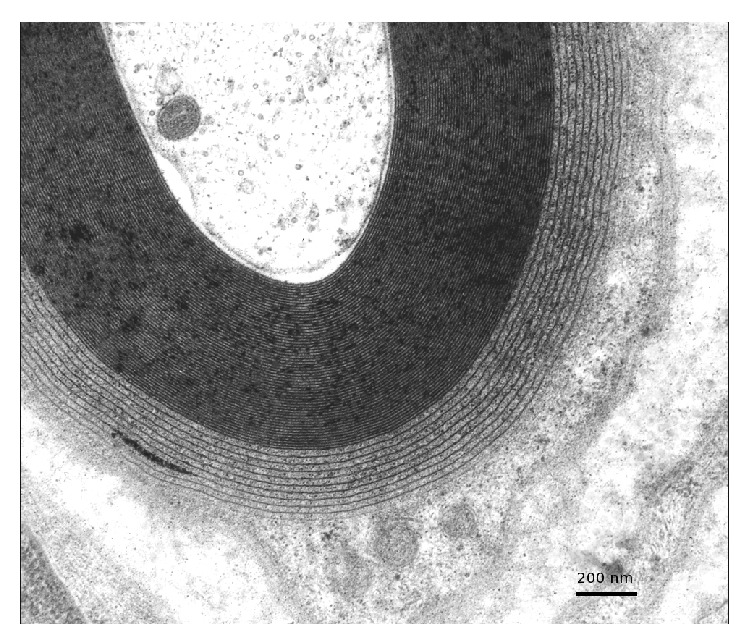
Typical widening of the most external myelin lamellae. Electron micrograph. Transverse section.

**Figure 2:**
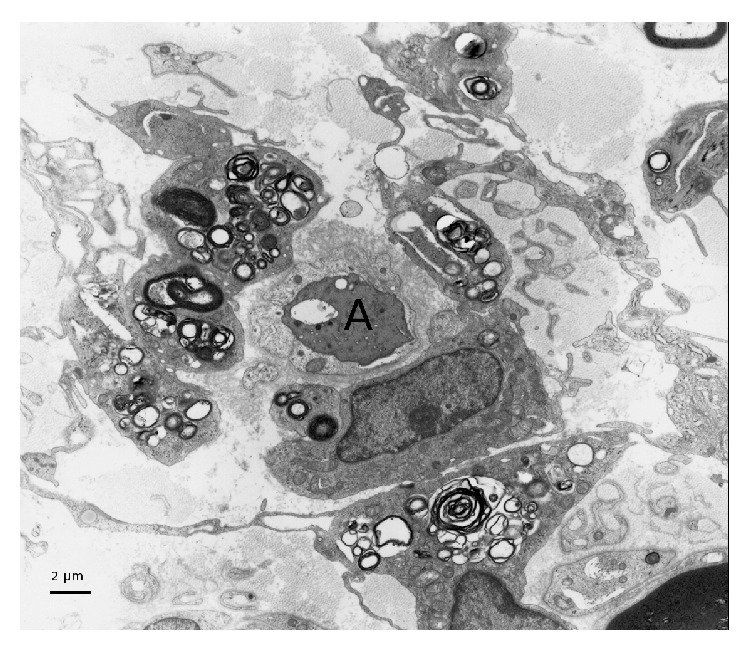
A naked axon (A) is surrounded by several macrophages loaded with myelin debris in a patient with the CIDP-like profile. Electron micrograph, transverse section.

**Figure 3:**
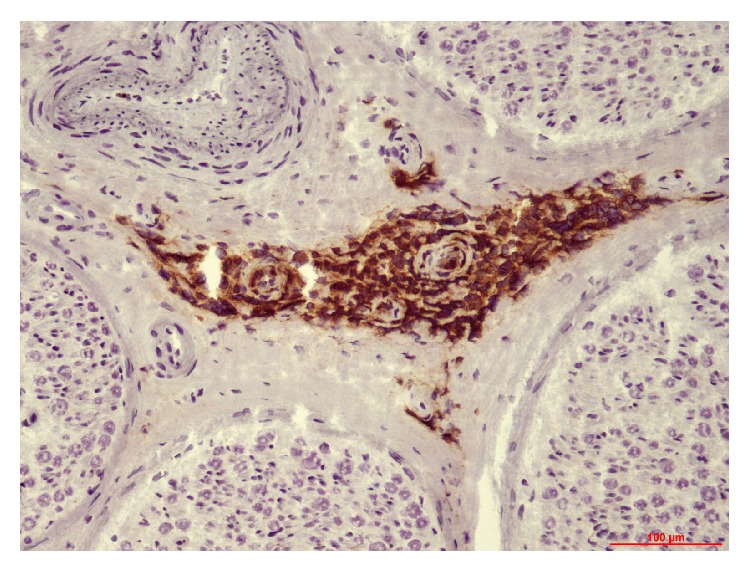
Several epineurial vessels are surrounded by prominent B cell infiltrates (patient 26). Paraffin transverse section labelled with anti-CD20 antibody.

**Figure 4:**
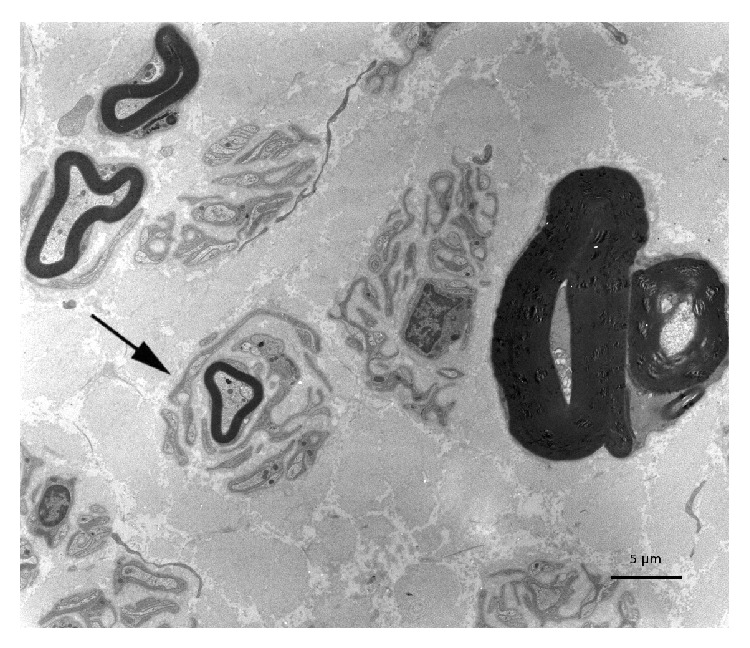
A myelinated axon is surrounded by onion bulb formations in a patient with the CIDP-like profile. Electron micrograph, transverse section.

Forty-three patients had undergone nerve biopsy. All nerve samples showed a decrease in myelinated fiber density on semithin sections. This axonal loss was highly variable among patients, ranging from slight to severe. Among patients who underwent nerve biopsy, 26 (60%) had typical widening of myelin lamellae (WML) on electron microscopic examination (Figure 1). WML usually concerned a minority (5–10%) of large myelinated axons. Most of these patients had mild cellular infiltrates composed of lymphocytes (anti-CD45 staining) and macrophages (anti-CD68 staining): 8 of these had macrophage-associated demyelination, a feature considered typical of CIDP (Figure 2). One of the female patients (26) with MGUS and WML had also prominent B cell infiltrates in the epineurium (anti-CD20 staining) suggesting the conversion of the gammopathy to a B cell lymphoma (Figure 3). Seventeen (40%) of the patients who underwent nerve biopsy had no WML. All of these patients had features typical of a demyelinating neuropathy with onion bulb formations and/or completely demyelinated axons (Figure 4). Pictures of remyelinating axons with abnormally thin myelin sheaths were frequent. Additionally, macrophage-associated demyelination was encountered in all nerve samples from these patients. Thirty-five patients had indirect immunofluorescence (IIF) to test reactivity of their serum against normal nerve. IIF failed to show any staining with anti-IgM and/or anti-C3D in 9 patients and was mostly negative in patients with the CIDP pattern and a low titer of anti-MAG antibodies. Nerve biopsy findings were not correlated with any clinical subtype as defined above.

### 3.5. Correlations according to the Electrodiagnostic and Neuropathological Status

The details of the two subpopulations of patients according to the electrodiagnostic pattern are summarized in Table 2. Among the 37 patients who had the typical anti-MAG pattern on nerve conduction studies, 27 had nerve biopsy, of which 21 (78%) showed typical WML (*p* < 0.05). Twenty-two of these 37 patients had IIF, which was positive in 20 (91%) cases. Of note, anti-MAG antibodies were above 10000 BTU in 32 (86%) of the 37 patients with NCS suggestive of anti-MAG neuropathy (*p* < 0.05).

Among the 17 patients who had nerve conduction studies consistent with CIDP, 12 underwent nerve biopsy, which showed histological abnormalities suggestive of CIDP, without WML on electron microscopic examination in 10 (83%) cases (*p* < 0.05). Ten of these 17 patients had IIF, which was negative in 7 cases. Moreover, 10 of these 17 patients had anti-MAG antibody titer below 10000 BTU (*p* < 0.05).

Four patients who had concordant nerve conduction and neuropathological findings suggestive of anti-MAG neuropathy had low titers of anti-MAG antibodies (1111–8663 BTU) but positive SGPG antibodies. IIF was carried out in 3 of these 4 patients and was positive in all.

## 4. Discussion

The objective of this retrospective study was to reexamine the concept that polyneuropathy associated with anti-MAG antibodies is a homogeneous entity. This syndrome is usually characterized by a slowly progressing sensory ataxic neuropathy. Nerve conduction studies usually show a symmetrical demyelinating neuropathy with distal accentuation and the phenotype is sometimes referred to as distal acquired demyelinating symmetric neuropathy (DADS) [16]. The demographic features of our patients (mean age at diagnosis of 69 years, sex ratio of 2.7 favoring males, and mean history of symptoms around two years before diagnosis) do not differ from previous studies [5]. Similarly, the chronic progression of symptoms we observed in our patients is a classic feature of this condition [4, 18].

In the present study, 53% of the patients had a clinical sensorimotor neuropathy and the presence of motor signs was not associated with the duration of the disease. We think this feature has not been emphasized previously. Indeed, in the follow-up series of Nobile-Orazio et al., disability was mainly caused by prominent ataxia, although motor signs were not mentioned [18]. Similarly, 28% of our patients had prominently sensory symptoms (without ataxia) that were sometimes painful suggesting small fiber involvement. We did not check for small fiber loss by skin biopsy, as this technique was not yet available in our center at the time of the study. Only 32% of our patients had prominent sensory ataxia. This is in contrast with previous studies where ataxia was found in more than two-thirds of patients [6, 19].

The pattern of nerve conduction abnormalities in anti-MAG neuropathy has been widely described in the literature and most authors have emphasized the distal accentuation of nerve conduction slowing [20, 21]. Moreover, consensus diagnostic criteria for anti-MAG neuropathy have been published and mostly rely on this feature along with severe reduction of SNAPs [11]. In our series, only two-thirds of patients had these typical electrodiagnostic features, while 30% of our patients had nerve conduction studies consistent with CIDP [12]. Moreover, most (83%) of the patients with the CIDP-like pattern who underwent nerve biopsy did not display the classic WML on electron microscopic examination.

Widening of myelin lamellae (WML) was present in 78% of the patients who had nerve biopsy and nerve conduction studies typical of the DADS phenotype. WML is usually observed in the outer myelin lamellae and is caused by dissociation of the intraperiod lines by the monoclonal component and is a specific feature of anti-MAG neuropathy. In our patients, WML constantly coexisted with macrophage-associated demyelination, a feature that is considered typical of CIDP although it has been described by others in patients with demyelinating neuropathy and monoclonal gammopathy [22]. One patient in this series had prominent B cell infiltrates in the epineurium, a feature that prompted a change in her treatment.

In this study, patients have been tested for the presence of anti-MAG antibodies by ELISA. Western blot (WB) analysis has long been the gold standard for detection of anti-MAG antibodies and positive WB is present in approximately 50% of patients with IgM monoclonal gammopathy and a demyelinating polyneuropathy [23]. The ELISA method, which is much simpler, can be applied in most immunology laboratories. Moreover, a recent work has shown that it is valid and robust in detecting anti-MAG antibodies in patients with IgM MG and polyneuropathy [13]. In this series, the ELISA test with a cut-off value of 1500 BTU was proven more sensitive than WB (71.2% versus 54%) to detect anti-MAG antibodies in patients with IgM MG and a demyelinating polyneuropathy. In the present study, we used a cut-off value of 1000 BTU but only 2 patients had a titer below 1500 BTU (see Table 1) so we think this has not significantly biased our results.

Results of indirect immunofluorescence were highly correlated with electrodiagnostic studies and neuropathological data in our patients. Indeed, among the ten patients who had an indirect immunofluorescence study in the CIDP group, the test was negative in seven. This was also correlated with a low titer of anti-MAG antibodies, which was below 10000 BTU in 10 out of 17 patients in this subgroup. This feature was not surprising, as other authors have previously shown that sera from patients with low titers of anti-MAG antibodies did not bind normal nerve, suggesting microheterogeneity of anti-MAG antibodies [24]. Conversely, the majority of patients with neurophysiological studies consistent with anti-MAG neuropathy and high titers of anti-MAG antibodies had a positive indirect immunofluorescence study. This simple test has been reported as highly specific even in patients with no detectable IgM gammopathy [25].

Four patients (11, 18, 23, and 29) in our series had a unique immunopathologic profile. They had a low titer of anti-MAG antibodies by ELISA (1111 to 8363 BTU) and 2 of these had a negative Western blot. These 4 patients, who had electrodiagnostic testing suggestive of anti-MAG neuropathy and typical WML for 3 of them, had positive SGPG antibody testing. This may suggest that anti-SGPG antibody testing is more sensitive to detect a relationship between the polyneuropathy and IgM monoclonal gammopathy in some patients, as previously outlined [26]. Moreover, in the recent series by Kuijf et al., the positivity of anti-MAG antibody and anti-SGPG antibody testing was highly correlated but 4 patients with a demyelinating neuropathy and IgM gammopathy had negative anti-MAG antibodies and positive anti-SGPG antibodies [13].

In our patients with high (>10000 BTU) titer of anti-MAG antibodies, nerve conduction studies were almost constantly suggestive of the DADS phenotype although the clinical picture was heterogeneous. Moreover, most of these patients had positive indirect immunofluorescence and WML. These data point to a direct relationship between the monoclonal gammopathy and the demyelinating neuropathy, by contrast with some of the patients with the CIDP-like profile. Additionally, 5 patients had positive anti-MAG antibodies by immunochromatography without quantification and could not have ELISA testing. Nevertheless, 4 of these patients had positive indirect immunofluorescence and 4 had nerve biopsy showing positive direct immunofluorescence and the presence of WML.

In summary, the data presented here clearly show the heterogeneity of polyneuropathy associated with anti-MAG antibodies. The clinical and pathological heterogeneity has already been emphasized recently [27], but we expand previous findings by demonstrating relationships between immunological, neurophysiological, and pathological profiles. In our series, clinical analysis suggests three main patterns of peripheral nervous system involvement: a sensory ataxic neuropathy, an almost purely subjective sensory (sometimes painful) neuropathy, and a sensory and motor neuropathy with variable degrees of weakness. Similarly, electrodiagnostic studies revealed that most of the patients had the phenotype referred to as DADS, although at least one-third of the patients had a CIDP-like presentation that may indicate a different pathophysiologic mechanism. Altogether, our data suggest that patients with anti-MAG neuropathy based on ELISA testing may be divided in two subgroups, according to nerve conduction studies, nerve biopsy findings, immunofluorescence data, and titers of anti-MAG antibodies. The first subgroup consists of patients with the typical anti-MAG neuropathy who have a sensory or sensorimotor polyneuropathy with demyelinating features predominating in the distal segments of nerves, prominent alterations of sensory potentials, positive immunofluorescence studies, WML, and high titers (above 8–10000 BTU) of anti-MAG antibodies. The second subgroup consists of patients with a CIDP-like neuropathy, who have a sensory or sensorimotor neuropathy with demyelinating features involving intermediate or proximal segments of nerves, sometimes preserved sensory potentials, negative immunofluorescence studies, no WML, and low titers of anti-MAG antibodies.

As the present study was retrospective, the phenotypes defined here were not taken into account at the time the patients were treated, and the care of the patients studied was considerably heterogeneous, from supportive treatment alone to the use of intravenous immunoglobulin, immunosuppressants, or monoclonal antibody (rituximab). We expect that our findings may have implications regarding the choice of therapy. Indeed, as reviewed [28], immunomodulatory treatments like intravenous immunoglobulin (IVIg) have demonstrated a short term and modest effect on patients with polyneuropathy and anti-MAG antibodies and are not usually considered as a first-line treatment of this condition [29]. Moreover, case reports have emphasized that a motor phenotype of anti-MAG associated neuropathy could be linked to a good response to IVIg [30]. Similarly, open-label trials have shown that plasma exchange in combination with cyclophosphamide may be beneficial in the short term [31] but does not provide any advantage over chlorambucil alone [32]. More recent studies suggest that rituximab is effective but in no more than half of the patients with polyneuropathy associated with anti-MAG antibodies [33, 34]. We think these discrepancies may be partly due to the heterogeneity of this syndrome, which may involve different pathogenic mechanisms. Deciphering these mechanisms is challenging but may help finding the best targets to treat patients with a potentially disabling disease.

## 5. Conclusions

We have described a series of patients with polyneuropathy associated with IgM monoclonal gammopathy and anti-MAG antibodies. Anti-MAG neuropathy is regarded as a homogeneous disorder characterized by a sensory ataxic neuropathy sometimes associated with tremor [5], but our personal experience is that this syndrome is characterized by more heterogeneity than was previously reported.

This study clearly demonstrates that anti-MAG neuropathy is indeed a relatively heterogeneous entity and that this heterogeneity can be found at the clinical, neurophysiological, and pathological levels. We suggest that this heterogeneity may be underlined by different pathophysiological mechanisms that remain to be elucidated. If this assumption is true, it could have major therapeutic implications in this frequent and sometimes debilitating disorder.

## Figures and Tables

**Table 1:** Main clinical and laboratory data in 60 patients with polyneuropathy, IgM gammopathy, and anti-MAG antibodies.

Patient	Age	Gender	Duration of symptoms (months)	Clinical picture	Limb involvement	NCS findings	Type of gammopathy	Anti-MAG titre (BTU)	DIF	IIF	Other antibodies	EM pattern
1	74	M	84	SM	D, Sy, 4L	MAG	MGUS	26 870	IgM, C3d	IgM, C3d	Neg	WML
2	69	F	36	A	D, Sy, 4L	MAG	MGUS	61 186	IgM, C3d	IgM, C3d	Neg	CIDP
3	63	F	24	A	D, Sy, 4M	MAG	Spleen lymphoma	23 800	ND	IgM, C3d	Neg	ND
4	64	F	18	S	D, Sy, 4L	MAG	MGUS	15 000	IgM, C3d	IgM, C3d	Neg	CIDP
5	76	M	12	A	D, Sy, 4L	MAG	MGUS	70 000	ND	ND	Neg	ND
6	75	M	204	SM	D, Sy, LL	MAG	Waldenström	57 237	IgM	IgM, C3d	Neg	WML
7	66	M	108	SM	D, Sy, 4L	SA	MGUS	48 584	Neg	Neg	Neg	WML
8	78	M	108	A	D, Sy, 4L	MAG	Hairy cell leukemia	14 125	Neg	Neg	Neg	WML
9	73	M	24	S	D, Sy, LL	MAG	MGUS	34 621	IgM, C3d	IgM, C3d	Neg	WML
10	74	F	36	S	D, Sy, 4L	MAG	MGUS	66 970	ND	ND	Neg	ND
11	75	M	60	SM	D, Sy, LL	MAG	Chronic myelomonocytic leukemia	1 312	Neg	IgM	SGPG/Gang	CIDP
12	70	F	24	SM	D, Sy, LL	ND	MGUS	70 000	ND	ND	SGPG	WML
13	57	M	12	S	D, Sy, LL	MAG	MGUS	Pos (WB)	ND	ND	Neg	WML
14	56	M	3	S	D, Sy, LL	MAG	MGUS	12 000	ND	ND	Neg	ND
15	61	M	18	SM	D, Sy, LL	MAG	MGUS	20 059	Neg	IgM	Gang/Sulf	CIDP
16	66	M	12	S	D, Sy, LL	ND	MGUS	55 127	Neg	ND	Neg	WML
17	69	F	36	A	D, Sy, LL	MAG	MGUS	70 000	ND	ND	ND	ND
18	74	M	24	SM	D, As, LL	MAG	MGUS	1 111	ND	ND	SGPG	WML
19	79	M	9	SM	P, As, LL	MAG	MGUS	19 127	IgM, C3d	IgM, C3d	SGPG	WML
20	60	M	192	SM	D, Sy, 4L	MAG	MGUS	76 210	Neg	Neg	Gang	WML
21	57	M	12	SM	D, As, 4L	MAG	MGUS	70 000	Neg	IgM	Neg	CIDP
22	46	M	12	SM	D, Sy, LL	ND	MGUS	23 915	Neg	IgM	SGPG/Gang	WML
23	58	M	11	S	D, As, UL	MAG	MGUS	1 794	IgM, C3d	IgM, C3d	SGPG	WML
24	66	M	12	SM	D, Sy, LL	MAG	MGUS	42 346	ND	IgM	Neg	ND
25	78	M	12	SM	D, Sy, LL	SA	Waldenström	97 194	IgM	IgM	Neg	WML
26	60	F	24	S	D, Sy, LL	MAG	Chronic lymphocytic leukemia	70 000	IgM, C3d	Neg	SGPG	WML (B cell inf)
27	69	M	96	A	D, Sy, LL	MAG	MGUS	Pos (WB)	IgM	IgM	Neg	WML
28	73	M	12	A	D, As, LL	MAG	MGUS	34 546	IgM	IgM	Neg	WML
29	72	M	24	SM	D, As, LL	MAG	MGUS	8 363	C3d	IgM, C3d	SGPG	WML
30	64	M	24	SM	D, Sy, 4L	MAG	MGUS	70 000	ND	ND	SGPG/Gang	WML
31	52	M	72	SM	D, Sy, 4L	MAG	MGUS	27 721	Neg	IgM, C3d	Neg	WML
32	75	F	12	S	D, Sy, 4L	MAG	MGUS	Pos (WB)	IgM	Neg	Neg	WML
33	67	F	6	S	D, Sy, LL	MAG	MGUS	35 971	ND	ND	Neg	ND
34	52	M	24	SM	D, Sy, LL	MAG	MGUS	16 272	ND	IgM, C3d	Neg	ND
35	57	F	96	SM	D, Sy, LL	MAG	MGUS	70 000	IgM, C3d	IgM	SGPG	WML
36	61	M	2	SM	D, Sy, LL	MAG	MGUS	Pos (WB)	ND	ND	SGPG/Gang	WML
37	57	F	24	S	D, Sy, 4L	MAG	MGUS	70 000	ND	ND	Neg	ND
38	78	M	72	S	D, Sy, LL	MAG	MGUS	13 395	IgM, C3d	IgM, C3d	Neg	WML
39	79	F	12	S	D, Sy, LL	MAG	MGUS	70 000	Neg	IgM	Neg	WML
40	73	M	144	SM	D, Sy, LL	MAG	MGUS	23 198	ND	ND	Neg	ND
41	71	M	12	S	D, As, LL	MAG	MGUS	4 165	ND	ND	ND	WML
42	87	M	24	SM	D, Sy, 4L	MAG	B cell lymphoma	70 000	Neg	IgM	Neg	WML
43	56	F	6	S	D, Sy, LL	ND	MGUS	56 644	ND	IgM	SGPG	ND
44	50	M	3	SM	D, Sy, 4L	CIDP	MGUS	61 960	ND	ND	Neg	ND
45	58	M	12	SM	D, As, LL	CIDP	MGUS	3 008	Neg	Neg	Neg	CIDP
46	77	M	48	SM	D, As, LL	CIDP	Waldenström	16 958	C3d	Neg	Neg	CIDP
47	67	M	18	SM	D, As, 4L	CIDP	MGUS	2 636	Neg	C3d	Neg	CIDP
48	77	M	3	A	D, Sy, LL	CIDP	MGUS	4 342	Neg	Neg	Neg	CIDP
49	65	M	48	A	D, Sy, 4L	CIDP	MGUS	9 224	ND	Neg	Neg	ND
50	50	M	12	A	D, Sy, 4L	CIDP	MGUS	5 177	Neg	Neg	SGPG	CIDP
51	82	M	6	SM	D, Sy, LL	CIDP	Waldenström	70 000	Neg	C3d	Neg	CIDP
52	78	M	12	SM	D, Sy, LL	CIDP	NHML	26 058	IgM, C3d	Neg	Neg	WML
53	56	M	4	SM	D, As, 4L	CIDP	MGUS	6351	Neg	Neg	Neg	CIDP
54	75	F	15	SM	P, Sy, LL	CIDP	MGUS	17 530	Neg	Neg	Neg	CIDP
55	49	M	12	S	D, Sy, 4L	CIDP	MGUS	5328	Neg	Neg	Neg	CIDP
56	47	M	24	S	D, As, 4L	CIDP	MGUS	62 978	ND	Neg	Neg	ND
57	68	M	8	SM	D, Sy, 4L	CIDP	MGUS	8 300	Neg	Neg	Neg	CIDP
58	83	F	18	SM	D, Sy, 4L	CIDP	MGUS	36 726	ND	IgM, C3d	Neg	ND
59	69	F	48	A	D, As, LL	CIDP	MGUS	10 064	ND	Neg	Neg	ND
60	77	F	12	SM	D, As, LL	CIDP	MGUS	2884	Neg	Neg	Neg	CIDP

A: prominent ataxia, As: asymmetric, D: distal distribution, DIF: direct immunofluorescence, F: female, Gang: ganglioside, IIF: indirect immunofluorescence, M: male, LL: lower limb predominance, MGUS: monoclonal gammopathy of undetermined significance, Neg: negative, NHML: non Hodgkin's malignant lymphoma, S: pure sensory neuropathy without ataxia, Sy: symmetrical, SA: sensory axonal polyneuropathy, SGPG: sulfate-3-glucuronyl paragloboside, SM: sensory and motor polyneuropathy, UL: upper limb predominance, 4L: four limb involvement.

**Table 2:** Main clinical and laboratory data from 2 subpopulations of patients with polyneuropathy and anti-MAG antibodies according to nerve conduction findings.

	Patients with the anti-MAG pattern *n* = 37	Patients with the CIDP-like pattern *n* = 17	Statistical significance
Typical widening of myelin lamellae	21/27 (78%)	2/12 (17%)	*p* < 0.05
Positive indirect immunofluorescence	20/22 (91%)	3/10 (30%)	*p* < 0.05
Anti-MAG antibodies >10 000 BTU	32/37 (86%)	7/17 (41%)	*p* < 0.05
Mean titer of anti-MAG antibodies (SD)	38410 (26626) *n* = 33	20560 (23097) *n* = 17	*p* < 0.05
